# Implementing a Robotic Hepatopancreatobiliary Program for New Faculty: Safety, Feasibility and Lessons Learned

**DOI:** 10.21203/rs.3.rs-4271384/v1

**Published:** 2024-04-24

**Authors:** Britney Niemann, Christopher Kenney, J. Wallis Marsh, Carl Schmidt, Brian A. Boone

**Affiliations:** Division of Surgical Oncology, Department of Surgery, West Virginia University; Division of Surgical Oncology, Department of Surgery, West Virginia University; Division of Surgical Oncology, Department of Surgery, West Virginia University; Division of Surgical Oncology, Department of Surgery, West Virginia University; Division of Surgical Oncology, Department of Surgery, West Virginia University

**Keywords:** hepatopancreatobiliary, robotic surgery, program implementation, career

## Abstract

**Background:**

Robotic surgery is increasingly utilized in hepatopancreatobiliary (HPB) surgery, but the learning curve is a substantial obstacle hindering implementation. Comprehensive robotic training can help to surmount this obstacle; however, despite the expansion of robotic training into residency and fellowship programs, limited data is available about how this translates into successful incorporation in faculty practice.

**Methods:**

All operations performed during the first three years of practice of a complex general surgical oncology-trained surgical oncologist at a tertiary care academic institution were retrospectively reviewed. The surgeon underwent comprehensive robotic training during residency and fellowship.

**Results:**

137 HPB operations were performed during the initial three years of practice. Over 80% were performed robotically each year across a spectrum of HPB procedures with a 6% conversion rate. Median operative time, the optimal metric for operative proficiency and evaluation for a learning curve, was similar throughout the study period for each major operation and below several reported optimized operative times. Major complications were similar across the experience and comparable to published series.

**Conclusion:**

Comprehensive robotic training in residency and fellowship as well as a dedicated, well-trained operative team allows for early attainment of optimized outcomes in a new HPB robotic practice.

## Introduction

Robotic surgery was first utilized in neurosurgery in 1985 [1]. Since that time, there have been significant advances in robotic technology allowing for technical advantages when compared to laparoscopy, including enhanced dexterity, three-dimensional vision fields, tremor elimination, and improved ergonomics. These advantages led to robotic utilization across a number of surgical subspecialities, including hepatopancreatobiliary (HPB) surgery. While initial studies demonstrated feasibility of the robotic approach, subsequent work suggested clinical benefits for the robotic approach in HPB procedures [2–8]. For instance, Zureikat et al. showed a reduction in major complications following robotic pancreaticoduodenectomy (RPD) compared to the open approach [7]. Similarly, two retrospective studies comparing robotic and laparoscopic major liver resections found lower conversion rates and lengths of stay in the robotic group [6, 8].

The overall use of robotic surgery has increased substantially in the last 10 years, but despite promising outcome data, there has been a slower rate of adoption in HPB operations [9, 10]. This is likely the result of the substantial learning curve associated with these complex operations. For example, the learning curve for RPD was initially reported to be between 40 and 250 cases [11, 12]. Since that time, formal robotic training programs have evolved, allowing earlier acquisition of skills [13–16]. In fact, we previously demonstrated that a dedicated robotic training program can substantially reduce the learning curve for RPD for both new and seasoned surgeons alike [13].

In addition to robotic training, many authors have described their experience throughout the implementation of a robotic practice in HPB surgery [17–20]. These studies reflect the experience of seasoned surgeons who incorporate robotic approaches into existing HPB practices. However, there is limited data available regarding the implementation of an HPB robotic program for new faculty immediately following training. Therefore, we aimed to describe the experience and lessons learned throughout the implementation of a new robotic HPB practice in order to provide guidance for future programs.

## Materials and Methods

### Case Selection and Surgeon Profile

After Institutional Review Board approval was obtained, all operations performed by a single complex general surgical oncology fellowship-trained surgeon with focus in HPB at an academic tertiary referral center were reviewed in a retrospective manner. Cases from the first three years of practice (September 2018 to December 2021) were evaluated. The surgeon underwent a formal robotic training program during fellowship including basic robotic skills training, video-based simulation, biotissue drills, and proctored cases [14, 15]. The surgeon had 50% protected time for translational research for the duration of the experience, which impacts the reported case volumes.

### Data Collection

Prior to surgery, all patients underwent a multidisciplinary evaluation. Surgical approach, including open or robotic, was determined based on patient factors such as vessel involvement and history of prior abdominal surgeries. Patient characteristics such as age, sex, Charlson comorbidity index (CCI), history of prior abdominal surgeries, and pathologic diagnosis were collected. Operative details including the type of operation, operative approach, conversions from robotic to open, estimated blood loss (EBL), and operative time were evaluated. Operations classified as HPB included pancreaticoduodenectomy, distal pancreatectomy, pancreatic debridement, total pancreatectomy, duodenal resection, bile duct resection, hepaticojejunostomy creation, cholecystectomy, hepatic resection, hepatic artery infusion (HAI) pump, and Strong’s procedure (for SMA syndrome). Operative time was defined as the time between skin incision and skin closure. Core members of the robotic HPB surgery team were defined from the larger cohort of circulating nurses, surgical technologists and physician assistants to determine robotic team utilization and report any difference in outcomes when core members were not present. Postoperative outcomes included hospital length of stay (LOS), readmissions, and mortality rates. The Clavien-Dindo classification system was used to categorize postoperative complications. Complications were reported for the ninety-day post-operative period.

### Statistical Analysis

All data was tested for normality using the D’Agostino and Pearson test. Non-normally distributed continuous data are reported as median (Interquartile Range (IQR)) and analyzed using the Kruskal-Wallis test. Categorical data are reported as frequency and analyzed using a Chi Square test. p < 0.05 was considered statistically significant.

## Results

### Patient Clinical Characteristics

**Figure 1:**
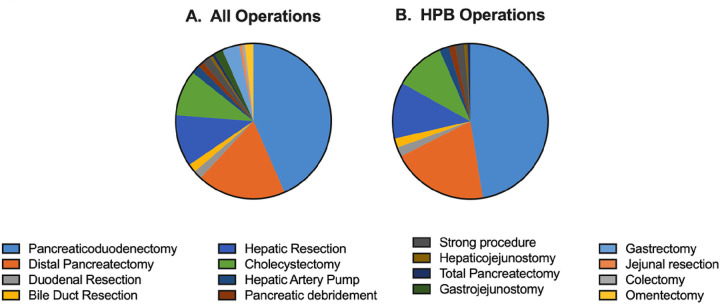
A wide operative breadth is feasible in an early HPB practice. The scope of practice during the first three years was examined. All operations performed (A) and HPB specific operations (B) are displayed.

A wide breadth of operations was performed throughout the first three years of practice. Of the 151 operations performed, 90% were primarily HPB (Figure 1). Patient demographics and clinical characteristics of patients undergoing HPB surgery are reported in Table 1. Patients were of similar age, sex, and CCI throughout the experience. Over 50% had a history of prior abdominal surgery across all 3 years with year 1 having 73%. Over two thirds of patients (64%) had a cancer diagnosis across all three years with specific diagnoses detailed in Table 1.

### Robotic Utilization and Versatility for HPB Procedures

**Figure 2:**
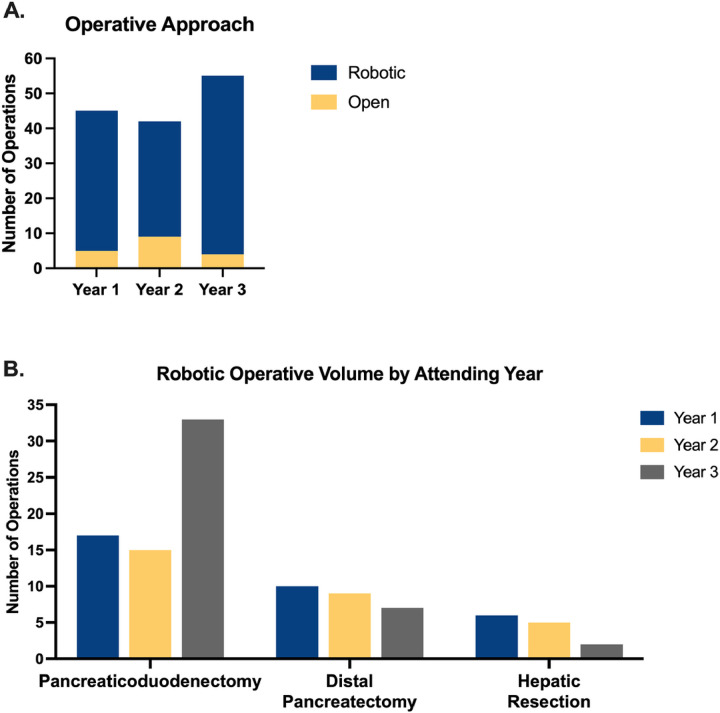
A robotic approach can be consistently utilized immediately following fellowship training. (A) All HPB cases during the first three years of practice are separated by operative approach, open or robotic. The majority of operations performed each year utilized a robotic approach. (B) A high volume robotic HPB practice is demonstrated beginning in year 1.

A substantial majority of cases, over 79%, were performed robotically each year with 93% performed with the robotic approach in year 3 (Figure 2 (a)). Six (13.3%) cases were converted to open the first year due to bleeding (n=2) or extensive adhesions in the setting of prior abdominal operations (n=4). Only one conversion to open occurred in both the second and third years. The most commonly performed HPB operation across all years was pancreaticoduodenectomy followed by distal pancreatectomy. The number of major HPB cases remained relatively constant over the three years (Figure 2 (b)). Furthermore, operations included a duodenal sleeve resection, lateral pancreaticojejunostomy (Puestow), distal pancreatectomy with celiac artery resection (Appleby), and Strong’s procedure were all performed robotically, demonstrating versatility of the platform for a variety of complex HPB procedures.

### Surgical Team

A second attending surgeon was present as the bedside assistant for the most critical portion of operation in over 64% of HPB cases, most commonly division of the pancreas neck and uncinate process attachments during pancreaticoduodenectomy. Pancreaticoduodenectomies and hepatic resections most commonly had at least 2 attending surgeons, up to 88% and 50%, respectively (Table 2). The core surgical team, including robotic HPB-dedicated circulating nurse, surgical technologist, and physician assistant was present for over 90% of cases.

### Peri-operative Outcomes

**Figure 3:**
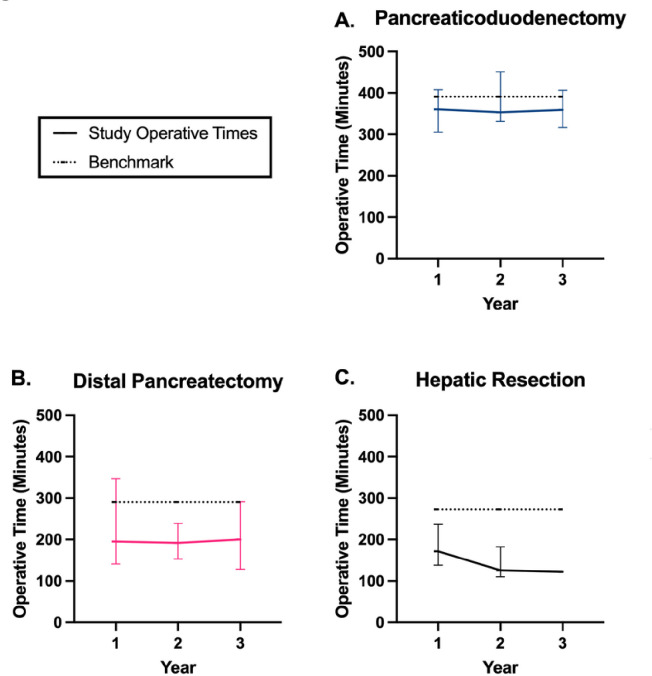
Median robotic operative times demonstrating absence of learning curve. Median operative times are a surrogate of surgical proficiency with established benchmarks demonstrating optimization. We found robotic operative times were below benchmark for three major HPB operations including (A) pancreaticoduodenectomy, (B) distal pancreatectomy, and (C) hepatic resection. Furthermore, operative times were consistent across the first three years of practice demonstrating little to no learning curve.

Clinical outcomes are listed in Table 2. Operative times for the most common procedures, including RPDs, distal pancreatectomies, and hepatic resections, as well as all HPB operations were evaluated as a surrogate of surgical proficiency (Figure 3).

There was no significant change in median operative time across the first three years for any operation, and all median operatives times fell below established benchmark operative times [8, 19, 21]. There was no significant difference between PD operative times using the robotic versus open approach. The median hospital LOS for all robotic HPB operations was 5 days each year. Readmissions within 90 days were found to be elevated during year 1 with 44% of all HPB patients requiring readmission, most common being after a pancreaticoduodenectomy (Table 2). Reasons for readmission after any operation in year 1 included superficial surgical site infection (SSI) (n=3), deep SSI (n=3), delayed gastric emptying (n=4), pancreatic leak (n=4), malfunctioning hepatic artery pump (n=1), fistula (n=1), and urosepsis (n=1). The readmission rate dropped to 15% and 16% in years 2 and 3, respectively. The 30- and 90-day mortality rates were 3% in the first two years and 0% and 4% in the last year, respectively (Table 2). Major complications from all operations ranged from 14% to 33%. Of note, the eight patients requiring a conversion from robotic to open had similar outcomes. Two (28%) patients requiring conversion were admitted within 90 days due to the development of delayed gastric emptying or deep SSI. Major complications were seen in 3 patients including one death.

## Discussion

Robotic surgery is becoming increasingly utilized in HPB surgery with an enlarging pool of evidence suggesting improvements in clinical outcomes [6–8]. A majority of results from HPB surgery come from established surgeons who implemented robotics into existing practices. Limited guidance is available to new faculty members with a focused practice utilizing the robotic platform in HPB surgery. Throughout the initial years of a new faculty practice, we found a wide breadth of HPB cases were feasible with the robotic platform, with perioperative outcomes that mirrored established benchmarks. These findings suggest that with the appropriate institutional environment and comprehensive training, safe and successful implementation of a robotic HPB practice is feasible.

Operative time is often used as the best measure of proficiency, as safely and efficiently moving through a complex operation suggests mastery of the technical skills necessary to complete the procedure. Studies investigating the learning curve for robotic surgery utilize this metric to monitor progression over time, often demonstrating a large decrease in operative times with early experience, followed by a more gradual plateau. The optimized benchmark for proficiency based on the last 100 RPDs from a series of 500 RPD operative times is 415 minutes [19]. In this study, we demonstrate a median operative time below this benchmark at 360 minutes during year 1. Similarly, the optimized operative time for a distal pancreatectomy has been reported as 290 minutes, whereas this study demonstrated an operative time of 195 minutes in year 1 [21]. Moreover, operative times were consistent across the first three years of practice, suggesting minimal learning curve throughout the experience. Average operative times for hepatic resections vary, but a recent meta-analysis showed an average operative time of 283 minutes [22]. During year 1 our operative time was 172 minutes with a small decrease seen during year 2.

Safety is of the utmost importance to all providers, but especially for junior faculty when starting a new practice. In this experience, we had low perioperative mortality and found the rate of major complications to be similar to previously reported outcomes. Additionally, there were no significant differences in complication rates between practice years. In particular, 24% of year 1 RPDs had a Clavien Dindo grade 3 or above complications, comparable to the 25% reported in a large series [19]. The same findings were seen with length of stay. However, 30 and 90-day readmission rates for all HPB robotic operations were elevated to 41% and 44%, respectively, during year 1 compared to 12.5% in subsequent years. The 30 and 90-day readmission rates for RPD during year 1, specifically, were both 47% with only 3 patients admitted for a Clavien Dindo complication of 3 or above. Larger series of RPDs have found 90-day readmission rates to range from 25 to 37 percent which is similar to those seen in years 2 and 3 in this study [11, 17, 19, 23]. These findings may suggest a heightened sense of alertness and greater degree of caution for a new faculty member, resulting in a lower threshold for readmission and may not be indicative of the robotic approach itself.

Patient selection is frequently cited as a key to success for early adopters of robotics. Previous authors have recommended the gradual implementation of robotics into a new HPB practice with cautious case selection [17]. However, with the advancement of formal training programs built into residency and fellowship programs, this may be less critical to early success. Over selection may limit the ability for patients to benefit from a minimally invasive approach when feasible and restricts the knowledge and experience gained from persevering through challenging cases. The current cohort includes a complex patient population with high rates of prior intra-abdominal operations, malignancy, and CCI present even in year 1, suggesting the avoidance of strict selection criteria for robotic utilization, even for junior faculty. This is important to acknowledge when interpreting our results. Our approach was to utilize the robot for as long as safely possible at the outset of an operation, even with a high index of suspicion for the eventual need to convert to an open procedure based on adhesive disease or vascular involvement. This approach resulted in successful robotic completion for several patients with prior major foregut surgery. Additionally, operating on patients with portal vein abutment prompted us to obtain complete vasculature control of the portal vein with laparoscopic bulldog clamps during the uncinate dissection very early in the experience (4^th^ RPD), which is a valuable technical skill to have for these procedures.

We believe several factors are critical to implementation of a successful robotic HPB surgery program especially for new attending surgeons, as demonstrated in the current work:

- **Comprehensive formal training in robotics during residency and fellowship –** this provides necessary technical skills, operative knowledge and prior faculty mentors who offer ongoing remote advice and support, all of which offer a strong foundation for future success.
- **Highly supportive department and institutional environment –** while only a handful of robotic HPB cases were done at the institution prior to 2018, the hospital already established programs in robotic cardiac, thoracic, head and neck, gynecologic and urologic surgery. New and experienced robot surgeon colleagues offered excitement and support of the program expanding to include HPB cases. The surgeon in this study was given dedicated robotic block time and a highly Functioning team.
- **Two Attending Approach –** while perhaps not required for success, we strongly encourage surgeons implementing a new robot HPB program to use a two-surgeon approach for the critical part of major procedures. The second attending offers technical help from more experienced hands during critical portions or challenging dissections, discusses details of the case as it unfolds, supports operative decision making, and represents a team approach for the culture of patient safety. Further, the bedside attending can effectively teach advanced laparoscopic skills to senior level residents and fellows much more directly than the other surgeon calling out instruction from the robot console.
- **Dedicated Robot HPB Operative Team –** a dedicated robotic operative team, including circulator and surgical assistant, is indispensable.[24] We are also fortunate to have robotic physician assistants on the surgical oncology team who are able to assist with patient positioning, docking and undocking, passing robotic instruments, and troubleshooting the robot itself.

This study does have numerous limitations. Importantly, this is a retrospective study examining the experience of a single surgeon at a single institution. These results may not be replicable across surgeons or institutions and should be interpreted with caution. The outcomes reported were the result of the surgeon being highly trained with a renowned, comprehensive robotics training program during residency and fellowship. Institutional support for the robotics program is critical to early success, and institutional resources and support vary significantly across centers. For instance, we utilized a two-surgeon approach for the majority of the major HPB cases, which may not be feasible at all hospitals. Furthermore, training resources for operative team members may differ. Despite these limitations, these results do provide a framework for safe and successful robotic surgery implementation.

Overall, we found the initiation of a new, predominantly robotic HPB practice was feasible with optimized operative times and complication rates seen within the first year of practice despite a complex and diverse patient population. We attribute this to the formal robotic training completed during residency and fellowship which allowed a diminished to absent additional learning curve during the initial years of practice. Additionally, substantial institutional support allowing for two faculty surgeon participation and a dedicated operative team is a critical component that made these results possible.

## Figures and Tables

**Table 1.** Patient demograhics

	Year 1 (N=45)	Year 2 (N=42)	Year 3 (N=55)	p value
**Age, median (IQR)**	60.8 (45.5-68.2)	64.1 (56.2-73.7)	61.4 (52.7-72.5)	0.13
**Female, n (%)**	27 (60.0)	21 (50.0)	21 (38.2)	0.09
**CCI, median (IQR)**	4.0 (2.0-5.5)	4.0 (3.0-6.0)	5.0 (3.0-6.0)	0.30
**Prior Abdominal Surgery, n (%)**	33 (73.3)	27 (64.3)	29 (52.7)	0.10
**Cancer Diagnosis, n (%)**	31 (68.9)	27 (64.3)	39 (70.9)	0.49
**Specific Diagnosis, n (%)**				
Gastrointestinal Stromal Tumor	1 (2.2)	0 (0)	1 (1.8)	
Peptic Ulcer Disease	0 (0)	1 (2.4)	1 (1.8)	
Duodenal Adenoma	0 (0)	1 (2.4)	0 (0)	
Duodenal Malignancy	1 (2.2)	2 (4.8)	2 (3.6)	
Pancreatic Ductal Adenocarcinoma	13 (28.9)	17 (40.5)	28 (50.9)	
PNET	6 (13.3)	3 (7.1)	4 (7.3)	
Premalignant Pancreatic Cysts	6 (13.3)	5 (11.9)	6 (10.9)	
Pancreatitis	4 (8.9)	3 (7.1)	0 (0)	
Benign Biliary	5 (11.1)	3 (7.1)	5 (9.1)	
Gallbladder Adenocarcinoma	2 (4.4)	0 (0)	0 (0)	
Cholangiocarcinoma	2 (4.4)	1 (2.4)	2 (3.6)	
Ampullary Adenocarcinoma	2 (4.4)	2 (4.8)	2 (3.6)	
Liver Metastases	1 (2.2)	4 (9.5)	2 (3.6)	
SMA Syndrome	2 (4.4)	0 (0)	2 (3.6)	

Abbreviations: (CCI) Charlson comorbidity index

**Table 2.** Clinical Outcomes for Robotic HPB Operations

	Year 1	Year 2	Year 3	p value
**≥2 attending surgeons**, n (%)				
Pancreaticoduodenectomy	16 (94)	15 (100)	31 (94)	0.62
Distal Pancreatectomy	4 (40)	3 (33)	2 (29)	0.88
Hepatic Resection	6 (100)	3 (60)	1 (50)	0.18
All HPB Operations	29 (73)	22 (67)	36 (71)	0.86
**Conversion to Open**, n (%)	6	1	1	NA
**EBL**, median mL (IQR)				
Pancreaticoduodenectomy	300 (50-550)	400 (150-800)	350 (175-775)	0.29
Distal Pancreatectomy	100 (43-400)	100 (50-150)	100 (10-300)	0.88
Hepatic Resection	200 (188-225)	40 (30-313)	150 (150-150)	0.33
All HPB Operations	100 (50-300)	150 (50-400)	200 (50-600)	0.60
**Hospital LOS**, median days (IQR)				
Pancreaticoduodenectomy	7 (6-10)	7 (5-15)	7 (5-9)	0.47
Distal Pancreatectomy	4 (4-5)	4 (4-5)	5 (4-5)	0.31
Hepatic Resection	5 (3-5)	2 (2-3)	4 (1-6)	0.21
All HPB Operations	5 (4-8)	6 (4-8)	5 (4-7)	0.76
**30-Day Readmission**, n (%)				
Pancreaticoduodenectomy	8 (47)	1 (7)	5 (15)	NA
Distal Pancreatectomy	3 (30)	1 (11)	0 (0)	NA
Hepatic Resection	2 (33)	1 (20)	0 (0)	NA
All HPB Operations	16 (41)	3 (9)	5 (10)	0.0002
**90-Day Readmission**, n (%)				
Pancreaticoduodenectomy	8 (47)	1 (7)	8 (24)	NA
Distal Pancreatectomy	5 (50)	2 (22)	0 (0)	NA
Hepatic Resection	2 (33)	1 (20)	0 (0)	NA
All HPB Operations	17 (44)	5 (15)	8 (16)	0.003
**30-Day Morality**, n (%)				
Pancreaticoduodenectomy	0 (0)	1 (7)	0 (0)	NA
Distal Pancreatectomy	0 (0)	0 (0)	0 (0)	NA
Hepatic Resection	0 (0)	0 (0)	0 (0)	NA
All HPB Operations	1 (3)	1 (3)	0 (0)	NA
**90-Day Morality**, n (%)				
Pancreaticoduodenectomy	0 (0)	1 (7)	2 (6)	NA
Distal Pancreatectomy	0 (0)	0 (0)	0 (0)	NA
Hepatic Resection	0 (0)	0 (0)	0 (0)	NA
All HPB Operations	1 (3)	1 (3)	2 (4)	NA
**Clavien-Dindo ≥ 3**, n (%)				
Pancreaticoduodenectomy	4 (24)	4 (27)	7 (21)	NA
Distal Pancreatectomy	4 (40)	1 (11)	0 (0)	NA
Hepatic Resection	2 (33)	1 (20)	0 (0)	NA
All HPB Operations	13 (33)	6 (18)	7 (14)	0.08

Abbreviations: (EBL) estimated blood loss; (LOS) length of stay
